# FedPneu: Federated Learning for Pneumonia Detection across Multiclient Cross-Silo Healthcare Datasets

**DOI:** 10.2174/0115734056333970241212132150

**Published:** 2025-01-02

**Authors:** Shagun Sharma, Kalpna Guleria, Ayush Dogra

**Affiliations:** 1 Chitkara University Institute of Engineering and Technology, Chitkara University, Rajpura, Punjab, India

**Keywords:** Federated learning, Computer-aided diagnosis, Pneumonia detection, Medical image visualization, Machine vision in medicine, FedPneu model

## Abstract

**Background:**

Pneumonia is an acute respiratory infection that has emerged as the predominant catalyst for escalating mortality rates worldwide. In the pursuit of the prevention and prediction of pneumonia, this work employs the development of an advanced deep-learning model by using a federated learning framework. The deep learning models rely on the utilization of a centralized system for disease prediction on the medical imaging data and pose risks of data breaches and exploitation; however, federated learning is a decentralized architecture which significantly reduces data privacy concerns.

**Methods:**

The federated learning works in a distributed architecture by sending a global model to clients rather than sending the data to the model. The proposed federated deep learning-based FedPneu computer-aided diagnosis model has been implemented in 2, 3, 4, and 5 clients architecture for early pneumonia detection using X-ray images. The key parameters configuration include batch size, learning rate, optimizer, decay, momentum, epochs, rounds, and random-split as 32, 0.0001, SGD, 0.000001, 0.9, 10, 100, and 42, respectively.

**Results:**

The results of the proposed federated deep learning-based FedPneu model have been provided in terms of round-wise accuracy, loss, and computational time. The highest accuracy of 85.632% has been achieved with 2-clients federated deep learning architecture, whereas, 3, 4, and 5 clients architecture achieved 85.536%, 76.112%, and 74.123% accuracies, respectively.

**Conclusion:**

In the proposed privacy-protected federated deep learning-based FedPneu model, the two-client architecture has been resulted as the most optimal framework for pneumonia detection among 3-clients, 4-clients, and 5-clients architecture. The model works in a collaborative and privacy-protected framework with a multi-silo dataset which could be highly beneficial for healthcare departments to maintain patient’s data privacy with improved prediction outcomes.

## 1 INTRODUCTION

Artificial Intelligence (AI) is a technology that includes machines and systems capable of performing tasks that typically require human intelligence, such as natural language understanding, image recognition, or data-driven decision-making [1, 2]. Machine Learning (ML) is a subset of AI that leverages algorithms to enable systems to learn and improve from experience without being explicitly programmed [3-5]. ML empowers machines to learn from data and make predictions based on that learning (6). Deep learning (DL) is also an AI-based learning method that significantly impacts data-driven and intensive applications in the vast healthcare domain, from smart agriculture to autonomous vehicles (4,7–11). The capability of DL in object detection using RetinaNet, semantic segmentation, and image classification with deep neural networks (DNN) has significantly impacted and surpassed the working mechanism and performance of traditional methods (12,13). In the medical domain, DL models are used for the patient's health monitoring and early identification of diseases using various datasets, including medical resonance imaging (MRI), tomography scans, electroencephalogram, electrocardiogram, and X-ray imaging (14–17). In MRI, X-rays, and CT scans, the DL models are capable of segmenting and penetrating the areas affected by the diseases. It thoroughly studies the body parts and creates internal segmented images of the affected area of the human body (18). These models can also be used in blood tests to diagnose various health challenges, such as detecting cancer, organ failure, and HIV diseases. In the recent past, the evolution of the COVID-19 pandemic has drastically changed the human living process and has vastly impacted economic growth all over the world. This pandemic brought up the necessity of having vaccinations and medications available. As per WHO, till June 2023, 6,945,714 deaths and 768,187,096 active cases were reported due to the pandemic [19]. However, these deaths have not been reported only due to COVID-19 but also due to the presence of both pneumonia and COVID-19 in the lungs. Pneumonia is a respiratory disease caused by high fever and cough leading to a lowering of oxygen intake and air sacs getting clogged with liquid or pus [2]. This disease can badly impact patients with weakened immune systems, infants, and the old age population. In the traditional healthcare scenario, the detection of respiratory diseases such as lung cancer, COVID-19, and pneumonia is diagnosed by performing chest X-ray imaging. However, expert advice from radiologists is required to detect the disease accurately. There are various pulmonary diseases that have similar symptoms which leads to difficulty in accurately identifying the disease. Additionally, when the patient is affected by more than one disease at a time, the diagnosing symptoms may overlap with other diseases, resulting in misdiagnosis and inappropriate medication. In such scenarios, the DL models, namely, RetinaNet and Mask-RCNN, are used for small-scale object identification and segmentation by creating anchor boxes on the overlapping objects, respectively [20]. The DL models are capable of performing highly accurate and precise disease prediction. However, the challenge is to collect patients' medical records at a centralized location, leading to data dependency, high computational resources, adversarial vulnerability, and data storage issues. The DL models need massive data to perform operations and predict the outcome. The schematic framework of DL is provided in Fig. (**1**). Healthcare is a vast domain that requires high privacy and data security due to legal rules and regulations implemented by the government. The sensitivity of the patient's data may make it impractical and illegal to transmit the data to a central server for disease prediction.

The healthcare industry has strict rules and regulations due to the Health Insurance Portability and Accountability Act (HIPAA), which imposes strict requirements for protecting the privacy and security of patient data. To resolve the data collection and privacy issues, Google introduced the term “Federated Learning” in 2016, which works in a decentralized manner and sends the weights updates to the central server instead of sending the client's data, as shown in Fig. (**2**) [21-23]. Apart from resolving the data collection challenges, the FL architecture can work on massively distributed, unbalanced, non-independent, and identically distributed data over edge computers [22, 24]. In this work, a FedPneu model has been implemented with the support of FL for the development of a reliable and authentic model architecture for pneumonia prediction (Table **1**).

The DL models are capable of extracting the most essential features from the images. These are highly efficient in disease detection; however, the challenge is to collect the dataset at a centralized location. This requires high bandwidth and memory for storing the large dataset at a single server. The FL models are used for the classification of diseases while also maintaining data privacy. These models use DL as the base model for implementation to securely identify the diseases in the images. The FL and DL models have different architectures in terms of data handling, storage, bandwidth, and flexibility, as tabulated in Table **1**.

This proposed work highlights the following contributions:

- The proposed work focuses on the development of a privacy-protected, reliable, computationally efficient, and authentic federated deep learning-based FedPneu pneumonia detection model using multi-client architecture.
- The proposed federated deep learning framework has been trained with independent and identically distributed data silos in 2, 3, 4, and 5 clients, and the updates have been aggregated using FedAVG aggregation technique. The proposed model resulted in the highest accuracy with 2-client architecture by configuring the learning rate as 0.0001, optimizer as SGD, epochs as 10, batch size as 32, and round value as 100.
- The collaborative and distributed architecture of the proposed federated deep learning-based model is highly optimal for preserving patients’ critical healthcare data and also resulting in optimal performance outcomes with the multi-silo datasets.

The proposed work is structured into several sections. Section II encompasses the integration of existing disease prediction models. Section III introduces the problem statement. Section IV provides detailed information about the dataset and the proposed methodology. Section V focuses on the evaluation of the proposed model, considering metrics, namely global accuracy, global loss, and computational time. Finally, Section VI draws the conclusion from the proposed work.

## 2 LITERATURE REVIEW ON VARIOUS DEEP LEARNING AND FEDERATED LEARNING FRAME-WORKS FOR DISEASE PREDICTION

This section discusses various existing disease detection models trained using DL and FL.

In [25] has been done, which is implemented for the prediction of COVID-19 pneumonia. The utilization of 3D CT scans was performed, and the images were resized to 512X512X160 to substitute for the network. The authors in [26] have introduced a ChexNet model, which works on the CNN architecture containing 121 deep layers. This model is trained on the chestX-ray14 dataset containing 100,000 CXRs of frontal views of the chest with the 14 different diseases. The pre-processing of the images has been performed to resize the images to 224X224 pixels for feeding the network. The hyperparameters, namely, optimizers of the model, have been set to 16, 0.001, and Adam for batch size, learning rate, and optimizer, resulting in an F1-score of 0.435. The model presented in [27] has been developed using VGG19, InceptionResNetV2, InceptionV3, DenseNet201, VGG16, Xception, MobileNetv2, and ResNet50 models for pneumonia identification. The models were implemented with the dataset containing 5,856 images of X-ray images and achieved the highest accuracy of 96.61% with the ResNet50 model, whereas VGG19, InceptionResNetV2, InceptionV3, DenseNet201, VGG16, Xception, and MobileNetV2 models achieved 85.94%, 96.09%, 94.59%, 93.66%, 86.26%, 83.14%, and 96.27%, respectively. In [28], the authors introduced an AlexNet model for pneumonia prediction. This model has been trained with SGD optimizers and 20 epochs with a dataset containing 3 different classes, namely, pneumonia, COVID-19, and normal lungs. The accuracy of the model has been achieved at 94.43% for pneumonia detection. In [29]resented a pneumonia detection model implemented using the InceptionV3 pre-trained model. This model was fine-tuned with the SGD optimizer and epoch count as 20 and achieved the highest accuracy of 97%. The model was further compared with the ML models, resulting in the proposed model as the most efficient and optimal for pneumonia detection.

In [30], the authors developed an FL framework by keeping the base models as ResNet18, COVID-Net, ResNext, and MobileNet for the detection of COVID-19 pneumonia in lung images. The hyperparameters, namely clients, epoch, batch size, learning rate, rounds, and weight decay for each model, have been kept the same as 5, 3, 0.00002, 100, and 0.0000001, respectively. The comparative results of these models have shown that the ResNet18 model outperforms, whereas MobileNet and COVID-Net have shown the same least value of loss. In [31], a capsule network-based FL model named COVID-CAPS has been proposed for the prediction of COVID-19 pneumonia in CXRs. This model has been proposed to resolve the drawbacks of the DL-based convolutional neural network (CNN) model in handling smaller datasets and providing better performance results. In [32], authors implemented the InceptionV3 model for performing multiclass classification to predict normal lungs, COVID-19, bacterial, and viral pneumonia. The dataset collected for the implementation contained 572 images, which were further augmented to increase the sample images for prediction. The results of the InceptionV3 model have been identified in AUC as 1 for binary classification for pneumonia and normal lung classification. In [33], various transfer learning models have been trained using FL, namely, MobileNetV2, ResNet18, DenseNet121, and ResNet50. For the prediction of pneumonia, authors have set three different optimizers *i.e*. AdaMax, Adam, and SGD+momentum. The results of the experiment were mentioned by performing the experiment in different optimizer scenarios, which identified that SGD+momentum with ResNet18 showed the highest performance. In [34], the authors proposed a FedHealth model FL model for the prediction of Parkinson's disease. The model was implemented with two different datasets, namely, droop and tremor. The results have shown that the proposed model has less accuracy of 74.9% in the dataset. Authors in [35], implemented a FL framework for the accurate identification of pneumonia in lung images. The VGG16, AlexNet, ResNet50, and CNN are used as feature extractors and classification base models. The results show that the VGG16 model outperformed all other models in terms of achieving optimal prediction outcomes. In [36], the authors implemented four pre-trained models, namely, ResNext50, convNet, AlexNet, and ResNet18, in the FL framework for predicting pneumonia in CXR images. The authors applied three different aggregation methods, namely, CoMed, GeoMed, and FedAVG. The results show that each model with different aggregation techniques depicts that FedAVG underperforms, whereas CoMed and GeoMed have shown the highest performance outcomes.

The authors in [37] implemented an FL framework by using VGG16 and ResNet50 techniques as the base models for the COVID-19 identification. To build this model, various hyperparameters, namely, batch size, learning rate, epochs, optimizer, and aggregation techniques, have been kept as 2, 0.001, 10, SGD, and FedAVG. An FL approach and centralized DL models have been proposed in [38] to predict the presence of COVID-19 in CXR images. In this work, the implementation was performed using the RetinaNet model, which identified that the model implemented in the FL environment shows outperforming results in comparison to centralized DL models. In [39], the authors proposed a centralized DL and FL approach for classifying COVID-19 and normal lungs in which the base model has been kept as VGG16. The client's values have been set to three hospitals. In this work, the hyperparameters such as rounds, aggregation methods, epochs, optimizer, and learning rate have been set as 10, FedAVG, 15, Adam, and 0.001, respectively. The results show that the FL framework implemented with the VGG16 pre-trained model has outperformed other models presented in the literature review. In [40], the authors proposed an FL framework that has been implemented using five different aggregation techniques, namely, FedBN, FedProx, FedNova, FedAVG, and SCAFFOLD. The implementation results identified that the FL approach implemented with FedBN outperforms, whereas FedNova shows the least accuracy at 5.57%. A decentralized FL approach has been proposed in [41] for COVID-19 and pneumonia prediction. For the implementation of this model, the authors used a particle swarm optimization technique applied to a CXR dataset collected from Kaggle. The results of this work have been identified for binary classification of pneumonia and normal lungs, and COVID-19 and normal lungs. In [42], authors proposed a FL framework, “DMFL_Net,” for multi-disease classification of lung cancer, pneumothorax, tuberculosis, and pneumonia. To implement this approach, the authors used DenseNet169 as the base model. To validate the performance of DMFL_Net, a performance comparison of DenseNet169 with VGG16 and VGG19 was done, which resulted in the proposed model outperforming.

Table **2** incorporates a detailed description of various DL models trained using FL in terms of the base model, hyperparameter tuning, performance results, research area, and research gaps.

## 3 PROBLEM STATEMENT

The objective of the proposed work is to develop an optimal and secured FL model for early and accurate pneumonia prediction. The problem statement of the proposed work has been divided into two parts: learning problem and optimization of the loss function. Further, the system model introduces the step-by-step procedure to create an FL framework for reducing loss function and achieving optimal performance outcomes.

### 3.1 Learning Problem

The learning problem of the proposed work is to minimize the value of the loss function by converging it towards the preset value of the threshold. Each client participating in the FL framework locally optimizes the loss function, and then the loss is converged globally by the global server. After round *R,* each client gets an optimal model called (w*) from the central server, as shown in the Eq. (**1**):


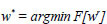
 (1)

Where *r* indicates 1,2,3,...R and *w^r^* is model weight at r^th^ round.

### 3.2 Optimization of Loss Function

The purpose of the proposed work is to minimize and optimize the loss function. The proposed FL model has batches of input images *I =* (*I_1_, I_2_, I_3_,…, I_N_*) and the label of each image as the prediction class *L =* (*L_1_, L_2_, L _3_,…, L _N_*). Minimizing the loss function leads to reduced error. In the proposed work, the consideration is that each hospital as the client has edge servers (*E_ser_*) and local datasets (*LD_ser_*) for the training of the model. A total number of “E” epochs have been used for the training of each *E_ser_*. The loss function of the local model at *E_ser_* can be computed using the Eq. (**2**):


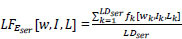
 (2)

Eq. (**2**) shows the loss of 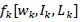 at *E_ser_*. *w* shows the updated value of weight.

*w_k_* shows the value of the updated parameter in different iterations at *E_ser_*.

The global loss function is generated at the global model updated after each round. Eq. (**3**) shows the global loss function.


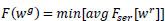
 (3)

Where r shows each round at *F_ser_* out of total rounds R.

### 3.3 System Model for Federated Learning Framework

The FL framework has various clients and a single server participating to create an optimal privacy-preserved prediction model. In this architecture, the model is trained locally using the client’s local dataset. The training of the local model at the client location reverts the parameter updates and gradients to the server instead of sending the actual data. The server uses a parameter aggregation technique to collect and average the local parameters of each client, which are used to update the global model. This updated global model is sent to each client for further training until it converges and achieves optimal performance.

#### 3.3.1 Model Training

This process includes the step-by-step procedure to train the global model at each client location with their local data, as shown in Fig. (**3**). The steps involved in the training process are as follows:

(1) Initially, the server creates a global model either by implementing pre-trained or transfer learning models on the large open-source image dataset.

(2) Each client “hospital” has its own local dataset of pneumonia and normal CXR images.

(3) Each hospital participating in the architecture downloads the global model, and the weight parameters and training of the global model are done by using local data at the client’s location. After the successful training, the model parameters updates, such as weight values and gradients, are sent back to the server instead of sending the local data to the central location.

(4) The server uses the FL aggregation technique to average the model weights from each client and creates another updated global model with the averaged parameter to create an optimal and privacy-preserved pneumonia detection model. In this, privacy is maintained throughout the complete framework, as neither the local dataset is shared with the peer clients nor with the central server.

(5) If the global model is not optimal, the server sends the model to each client again for the next round. Otherwise, the model is identified as ideal on the basis of achieved accuracy and error values in each round.

This FL-framework is decentralized and privacy-preserved as multiple clients participating in the architecture don’t have access to the private data of their peer clients. The server aggregates the weight values instead of collecting the data at the central location. This model enhances data privacy, especially for the data belonging to healthcare departments. The FL is a decentralized architecture in which the data is kept within the local devices and not shared with the central server for training. The initial model is shared with the clients for local training with their private datasets. It maintains transparency of the weight flow and prevents the raw data from unauthorized access in a privacy-preserving manner. In addition, the data also remains safe and preserved within the local clients as it is being processed and stored locally.

## 4 PROPOSED FL FRAMEWORK AND METHODOLOGY FOR PNEUMONIA PREDICTION

The proposed FL framework is used to predict pneumonia in CXR images. This framework has a central server *C_ser_ *) and clients *E_ser_ *) participating in the architecture. The proposed work uses hospitals as the *E_ser_* and trains the global model locally with their data (*LD_ser_ *) . In this architecture, the data is not sent to the central server, unlike traditional DL models. Thus, it creates secure communication between clients and a server, which leads to data security and optimal results.

### 4.1 FL framework for Local update at *E_ser_*

Algorithm 1 defines the procedure of local training in which each client parallelly performs local training using their local dataset (*LD_ser_ *) . After the training, the loss is calculated at each epoch (E), which then computes the best local model with the least loss function. In the proposed model, the stochastic gradient descent (SGD) has been used as the optimizer and has been used as the momentum for accelerating the SGD's performance. Further, the updated parameters, specifically weight values, are computed using equations (**4**) and (**5**). Lastly, the best local model was returned using equations (**6**) and (**7**).

**ALGORITHM 1: Local update at *E_ser_***

**Input:** *LD_ser_* , *E* , Batch size= *B*

**Output:** Model parameters *w_k_* , 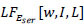


**Initialize:** 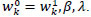



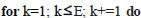



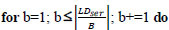


Calculating local update at *E_ser_* using SGD optimizer:


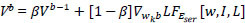
 (4)


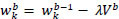
 (5)

Calculating the loss function using Eq. (**2**)

Selecting the local model for clients with


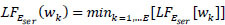
 (6)


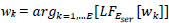
 (7)

→this will return the best local model

return *w_k_*, *LF*wk**

### 4.2 FL framework for Global Update at *C_ser_*

In this process, the global update and optimal model identification are done using algorithm 2. Each client trains the global model with their local dataset, and the aggregation of each parameter update collected from different clients has been done using the FedAVG model aggregation technique in the proposed work. After round r, different global models were created as *C_ser_* . Finally, these global models are compared using *argmin* function to find the indices with the minimum loss value. The model with the least loss and highest accuracy has been considered the optimal model for pneumonia prediction.

**ALGORITHM 2: Global update at *C_ser_***

**Input:** *C_ser_* = ( *C_ser1,_ C_ser2_ , …, C_serN_* ), and rounds = R

**Output:** Model parameters w ^*^ , global loss function(most optimal)= *F(w ^g^ )*

**Algorithm GLOBAL UPDATE AT *C_ser_***

**for** r = 1,2,3, …, R **do**

**for each** *C_ser _* ϵ *E_ser_* in parallel calculate: **do**

LOCAL_UPDATE_AT_ *E_ser_*

***w ^r^* = FedAVG** (Computing the average of updates of each client)

Send *w ^r^* to the *C_ser_* .

### 4.3 Dataset Description and Proposed Methodology for FedPneu Federated Learning Model

This section discusses the CXR dataset and proposed methodology for the FedPneu federated learning model.

#### 4.3.1 Dataset

The proposed model was implemented using three different open-source datasets collected from Kaggle. The first dataset contains 5,856 CXR images ( 43 ). The second dataset has 188 images of normal and pneumonia lungs collected from ( 44 ). The third dataset contains 5,857 images collected from ( 45 ). Dataset pooling was done to collect the highest number of images for model training. This step has resulted in 10,440 images, which have been further divided among different numbers of clients for 2 clients, 3 clients, 4 clients, and 5 clients FL architecture. These dataset distributions have been shown in Fig. (**4**-**7**).

#### 4.3.2 Proposed Methodology for FedPneu Federated Learning Model for Early Pneumonia Detection

The proposed FedPneu model uses the FL framework to predict pneumonia from CXR images, as shown in Fig. (**8**) . This technique focuses on the development of an FL architecture, where the multi-layer perceptron (MLP) is used as the base model. This model consists of three fully connected layers, where the ReLU activation function is used for the first two layers, and the SoftMax activation function is used for the last layer. Further, the building steps of the proposed model have been shown in Fig. (**9**) , and the description of the same is given below:

##### 4.3.2.1 Data Loading

To work with the image dataset, the CV2 package has been installed to read the CXRs. The images were unidentical; hence, they have been converted to standard 28*28*3 image size.

##### 4.3.2.2 Package Installation

Google has created various built-in libraries and packages for implementing DL and FL. In the proposed work, various packages have been installed as tabulated in Table **3**.

##### 4.3.2.3 Normalization

For normalizing the image dataset, each pixel of the CXRs has been divided by 255 to range them between 0-1.

##### 4.3.2.4 Creating Clients

A function has been used for creating a total of 100 clients. A numpy array list and label list were created with images and their labels, respectively. Each client has been named as clients_1, clients_2, clients_3, …, clients_N.

##### 4.3.2.5 Creating Data Shards

The data shard is a process in which the training data is divided into N number of parts. Each part is used as the data for each client. This process is only used in experiments because, in practical scenarios, each hospital is having their own data. The creation of the data shard should be in such a way that it is equal to the number of clients participating in the framework.

##### 4.3.2.6 Creating Weight Scaling Factor Function

This function was created to calculate the weight scaling factor for each client in the FL architecture. This function contains two parameters: the first parameter contains a dictionary of clients and their datasets, whereas the second parameter has the names of each client involved in the FL framework

##### 4.3.2.7 Applying Aggregation Technique

A FedAVG technique has been implemented to aggregate the local weights from different clients using the sum_scaled_weights function. This technique creates the averaged weights, which have been sent to the global model for update.

##### 4.3.2.8 Creation of MLP

This is a DL model which consists of one or more hidden layers. The proposed model has three fully connected layers. This model was created with a stack of three layers using a sequential function in which two dense layers with 200 filters and a ReLU activation function were used. In the initial layer of the MLP, the input shape was used as 50,176 due to the input image size being 224*224. The third dense layer depicts the output class using the Softmax function.

This model has been initially designed for image classification tasks. In the proposed work, this model has been utilized for pneumonia detection in CXR images. It has been named as a multi-layer perceptron as it contains multiple stacks of layers namely, a fully connected layer, which is used for sequential model building. The hidden layers in the network have been used for extracting abstract patterns from the provided images of normal and pneumonia lungs. The ReLU activation solves the problem of vanishing gradient for improving the performance of the model and learning the patterns faster. Further, the additional third layer has been named as output layer, which utilizes softmax activation for producing the classification outcomes. This activation converts the output into probability distribution among multiple classes and ensures that the output should collectively result in the value equal to 1. The probability score depicts the final class of each image inserted into the model. This model is capable of identifying hierarchical patterns from the dataset. The model has been implemented by the combination of ReLU and softmax activation functions, which ensures that the model is capable of extracting complex patterns as well as providing accurate outcomes. This model balances the classification performance and computational efficiency which makes it highly suitable for pneumonia detection.

Initially, this model has been supplied with the dataset that has been divided among training and testing as 90% and 10%, respectively. This dataset has been undergone through the pre-processing phase, namely binarizing the images. Further, the binarized images have been undergone through the layer of the MLP model for feature extraction and classification. The hidden layers perform feature extraction and the output layer provides the classification outcomes in terms of accuracy, precision, recall, F1-score, and computational time.

##### 4.3.2.9 Randomizing the Data

The data needs to be divided among the chosen number of clients in the FL framework. This data was randomized using the shuffle function and distributed among different clients participating in the process.

##### 4.3.2.10 Binarizing the Images

The images have been transformed to convert them into binary form in a matrix where all the variables are indicated in an attribute, as shown in Fig. (**10**) . Later, the training and testing data split was done with a ratio of 90:10.

##### 4.3.2.11 Applying One-hot Encoding

This process has been used in the proposed work to depict the categorical variable in the numerical form. Both data from training and testing have been implemented with one-hot encoding by using the to_categorical function.

##### 4.3.2.12 Choosing the Number of Clients to Participate in the FL Framework

This step has been implemented to choose the number of clients for active participation in the FL framework out of all the clients associated with the central server. In the proposed model, the framework has been applied to two, three, four, and five clients' FL architecture. Furthermore, tf.data.Dataset. from_tensor_slices has been used to process and batch the test data.

##### 4.3.2.13 Hyperparameter Tuning

In the proposed FedPneu model, various hyperparameters, namely learning rate, communication_rounds, optimizer, decay, and momentum, have been tabulated in Table **4**.

##### 4.3.2.14 Initializing the Global Model

The global model has been initialized with the implementation of the FedPneu model using MLP with the two output classes as, pneumonia and normal lungs.

##### 4.3.2.15 Commencing the Global Training Loop

The process of implementing the FL framework has been started by using the weights of the global model to be set as the initial weights for local models. To get these weights, the proposed model has been implemented with global model.get_weights function. Afterward, a scaled_local_weights_list was created to collect the local model's weights by implementing the global model with the local dataset. This process works in iterations in which each client results in local updates called weights, which have been further substituted for the central server for aggregation and model update.

##### 4.3.2.16 Clearing the Memory

After each communication round, to reduce memory usage, the K.clear_session() function has been used to clear the used memory.

##### 4.3.2.17 Testing the Global Model

After FedAVG, the global model is tested using a test dataset, and the performance is measured in global accuracy, global loss, and computational time, which is then saved for the current iteration. However, the same procedure is followed until the optimal model with the highest global accuracy and lowest global loss has been found.

## 5 RESULTS AND DISCUSSION

This section discusses the results of the proposed FedPneu model for the prediction of pneumonia and normal lungs. The results of the model have been provided in global accuracy, global loss, and computational time.

### 5.1 Performance Results of the Proposed FedPneu Model with Client Value Set to Two

This section introduces the global accuracy, global loss, and computational time taken by the proposed FedPneu model for each communication round when the number of clients has been set to two.

Figs. (**11**-**15**) depict the graph of achieved global accuracy with respect to number of rounds varying from 1-20, 21-40, 41-60, 61-80, and 81-100, respectively. These figures show that with the increase in the number of rounds, the global accuracy also increases. The highest accuracy was achieved at round 100 at 85.632%; however, the accuracy at round 1 was identified at 57.763%. The change in the accuracy rate shows that when the number of rounds for training the model increases, the accuracy also starts to increase.

Fig. (**16**) depicts the global accuracy rate after each ten rounds. This graph depicts the achieved accuracy values along with an increase in the number of rounds. The figure shows that the accuracy increases at each round from 10-70; however, after round 70, the accuracy has been decreased in round 80 to 82%. Further, this accuracy has again started to increase and reached 83.178% and 85.632% at round 90 and round 100, respectively.

Fig. (**17**) depicts the global loss of the proposed FedPneu model while the client value has been set as two. This graph shows that there is a huge fluctuation in the global loss with the increase in the rounds. The lowest loss was identified in round 27 as 0.6882; however, the highest loss was identified in round 1 as 0.7912.

Table **5** presents the value of loss after each 10 rounds starting from round 5. The loss value has depicted that with the increase in the number of rounds, the loss has started to drop; however, in some rounds, this loss has been analyzed as high in comparison to the loss value in round 5.

Fig. (**18**) depicts the global loss graph after each of the 10 rounds. This graph shows that there is almost equal loss value at round 10, 20, 30, 40, 50, 60, 70, 80, 90, and 100 as 0.6911, 0.6912, 0.6911, 0.691, 0.691, 0.6917, 0.6885, 0.691, 0.6899, and 0.6885, respectively.

Fig. (**19**) shows the graphical representation of computational time with respect to the number of rounds. This graph illustrates that there is a huge fluctuation in the computation time with respect to an increase in the number of rounds. However, the highest computational time was taken by round 1 at 6.33 minutes, which is quite high. Furthermore, round 100 also took 5.27 minutes to complete, which is also high compared with the computational time of round 2 to round 99.

Fig. (**20**) represents the computational time taken by rounds 10, 20, 30, 40, 50, 60, 70, 80, 90, and 100 to train the model. Among all the rounds, the highest time was taken by the 100th round, which had a value of 5.27 minutes. However, round 70 has also taken 5 minutes to complete the training.

Figs. (**21**-**25**) show the graph of computational time with respect to number of rounds varying from 1-20, 21-40, 41-60, 61-80, and 81-100, respectively. These graphs illustrate that there is a huge fluctuation in the computational time taken by each round. The highest computational time of 6.33 minutes was taken by round 1, whereas the lowest computational time of 0.01 minutes was taken by round 79.

Fig. (**26**) shows the total computational time taken by rounds 1-20, 21-40, 41-60, 61-80, and 81-100 as 54.32, 49.5, 47.29, 47.32, and 51.41 minutes, respectively, which results that the highest computational time has been taken by rounds 1-20 to train the proposed FedPneu model.

### 5.2 Performance Results of the Proposed FedPneu Model with Client Value Set to Three

This section introduces the global accuracy, global loss, and computational time taken by the proposed FedPneu model for each communication round when the number of clients has been set to three.

Figs. (**27**-**31**) show the achieved global accuracy with respect to number of rounds varying from 1-20, 21-40, 41-60, 61-80, and 81-100, respectively. These figures illustrate that when the number of rounds has been increased, the global accuracy also starts to increase. In the implementation of the proposed FedPneu model with three clients, the global accuracy at round 1 was identified as 63.221%, which continuously increased till round 5. In rounds 6 and 7, the accuracy decreased and reached 55.672% and 49.558%, respectively. Further, at round 8, the accuracy started to increase till round 15 and reached 60.002%. With the increase in the number of rounds, the global accuracy reaches 81.009% at round 60. After the implementation of the 100^th^ round, the highest accuracy value of 85.536% was achieved.

Fig. (**32**) shows the global accuracy of the proposed FedPneu model with three clients after each 10 rounds. This graph depicts an increase in the global accuracy after 10 rounds, and the highest accuracy was achieved at round 100 at 85.536%.

Fig. (**33**) shows the global loss of the proposed FedPneu model with respect to the number of rounds. The graph shows that in the initial phase of the training, the loss was identified as 0.6912 for round 1; further, the loss started to increase and fluctuate. In round 100, the least loss of 0.6895 has been identified.

Table **6** shows the global loss of the proposed model starting from round 5. The value of the global loss at round 5 has been identified as 0.6909 and at round 100 as 0.6933. This table shows the loss value at different numbers of rounds, after each 10 rounds starting from round 5.

Fig. (**34**) depicts the global loss value after every 10 rounds. The graph depicts that among all these rounds, the minimum loss was identified at round 10 as 0.6903, and the highest was at round 100 as 0.6995.

Fig. (**35**) shows the computational time taken by the proposed model to complete the training at each round. The highest computation time was taken by round 9 at 10.18 minutes, and the lowest time was taken by round 46 at 0.21.

Fig. (**36**) depicts the computational time taken by rounds 10, 20, 30, 40, 50, 60, 70, 80, 90, and 100 for training the proposed FedPneu model. Among these rounds, the highest computational time was taken by round 70 at 8.07 minutes.

Figs. (**37**-**41**) show the graph of computational time for training the proposed FedPneu model with respect to number of rounds varying from 1-20, 21-40, 41-60, 61-80, and 81-100, respectively. This graph shows that the highest computational time was identified at round 11 as 10.18 minutes, whereas the least computational time was taken by round 46 as 0.21 minutes.

Fig. (**42**) shows the total computational time taken by rounds 1-20, 21-40, 41-60, 61-80, and 81-100, which resulted in the highest computational time has been taken by rounds 1-20 as 84.43 minutes and the lowest computational time has been taken by rounds 21040 as 44.48 minutes to train the proposed FedPneu model.

### 5.3 Performance Results of the Proposed FedPneu Model with Client Value Set to Four

This section introduces the global accuracy, global loss, and computational time taken by the proposed FedPneu model for each communication round when the number of clients has been set to four.

Figs. (**43**-**47**) depict the graph of achieved global accuracy of the proposed FedPneu model with four clients architecture with respect to number of rounds varying from 1-20, 21-40, 41-60, 61-80, and 81-100, respectively. These graphs show that with the increase in the number of rounds, the global accuracy starts to increase. In round 1, the accuracy starts from 47.541% and reaches 53.449% in round 10. This happens because of the deep training of the model and the increase in the data. This accuracy is achieved as 60.902% and 68.335% at rounds 20 and 40, respectively. Furthermore, the accuracy remains the same till round 56; however, at round 57, it reaches 68.433%. Later on, at round 80, an accuracy of 71.111% was achieved, and in round 100, it reached its peak with the highest value of 76.112%. Round 100 shows the highest value of accuracy, resulting in an optimal model for pneumonia prediction.

Fig. (**48**) depicts the global accuracy of the proposed FedPneu model after each 10 rounds. The results conclude that in the initial phase till round 10, the accuracy starts increasing and reaches 53.449%. When the number of rounds was increased, the global accuracy also started to increase till round 40 and reached 68.335%. At around 50 and 60, the accuracy remained constant at 68.335%. Further, the increase in the round has impacted the accuracy and resulted in better performance by achieving 70.005%, 71.111%, 74.771%, and 76.112% accuracies at rounds 70, 80, 90, and 100, respectively.

Fig. (**49**) represents the graph for global loss v/s number of rounds. With the increase in the number of rounds, a decrease in the global loss has been observed. At rounds 10, 20, 30, 40, 50, 60, 70, 80, 90, and 100, this loss has been measured as 0.6931, 0.673, 0.6936, 0.6983, 0.6045, 0.6842, 0.6876, 0.6686, 0.5826, and 0.5266, respectively. The values of the loss have shown that the number of rounds is inversely proportional to the global loss, which equates that with the increase in the rounds, the loss function starts to decrease, and round 100 shows the least value of global loss. This shows that the optimal FedPneu model was achieved at round 100 with the least loss value.

Table **7** depicts the computed values of global loss of the proposed FedPneu model for the prediction of pneumonia in CXR images. The change in the global loss shows that at round 5, the global loss is 0.6334, whereas at round 15, it has been calculated as 0.631, which is less in comparison to round 5. Later on, it was identified that with the increase in the number of rounds, the global loss decreases. Furthermore, this loss increased in round 65 and again decreased in round 75 to 95 and reached 0.5546.

Fig. (**50**) shows the global loss of the proposed FedPneu model after each 10 rounds. These results depicted that with the increase in the number of rounds, the global loss started to decrease by resulting the loss values as 0.6931, 0.673, 0.6936, 0.6983, 0.6045, 0.6842, 0.6876, 0.6686, 0.5826, and 0.5266 at round 10, 20, 30, 40, 50, 60, 70, 80, 90, and 100, respectively.

Fig. (**51**) illustrates the computational time taken by the proposed FedPneu model to complete each round. This graph shows that the computational time is very high in the first round as compared to the 100th round. However, the least and highest computational time has been taken by round 33^rd^ at 0.44 minutes and round 57^th^ at 10.33 minutes.

Fig. (**52**) depicts the computation time of the proposed FedPneu model after each 10 rounds. The results identified that the computation time for each round varies and resulted in the time in minutes as 3.45, 6.05, 3.19, 4.51, 4.56, 4.56, 6.02, 3.47, 7.08, and 1.49 minutes for implementing round 10, 20, 30, 40, 50, 60, 70, 80, 90, and 100, respectively.

Figs. (**53**-**57**) depict the comparison of computational time taken by various rounds varying from 1-20, 21-40, 41-60, 61-80, and 81-100, respectively for the implementation of the proposed FedPneu model with four clients architecture. The highest computational time was taken by round 57 at 10.33 minutes, and the lowest computational time was 0.44 minutes by round 33.

Fig. (**58**) compares the total computational time taken by the rounds between 1-20, 21-40, 41-60, 61-80, and 81-100. This comparison has resulted in the highest computation time of 113.16 minutes and lowest computational time of 65.47 minutes, which has been taken by rounds 41-60 and rounds 21-40, respectively. Additionally, to complete the entire implementation of the proposed FedPneu model with four client architectures, a computational time of 426.22 minutes was found, which is very high in comparison to traditional DL models.

### 5.4 Performance Results of the Proposed FedPneu Model with Client Value Set to Five

Figs. (**59**-**63**) depict the achieved global accuracy with respect to number of rounds varying from 1-20, 21-40, 41-60, 61-80, and 81-100, respectively for the proposed FedPneu model implemented with five clients architecture. These graphs depict that there is an increase in the accuracy with the increase in number of rounds. The lowest accuracy was identified at round 1, while the highest accuracy of 74.123% was achieved at round 100.

Fig. (**64**) shows a global accuracy achieved by the proposed FedPneu model for pneumonia prediction after every 10 rounds. The results show that the highest accuracy among these rounds was achieved in round 100 at 74.123%, whereas the accuracy in round 10 was 52.109%.

Fig. (**65**) represents the global loss of the proposed FedPneu model while implementing it with five clients. It depicts that there is a huge fluctuation in the global loss with the increase in the number of rounds. The highest global loss has been identified at round 100 as 0.7711.

Table **8** shows the global loss identified at different rounds. This shows that the highest global loss was identified at round 5, which reached 0.7106 at round 95.

Fig. (**66**) illustrates the global loss identified at rounds 10, 20, 30, 40, 50, 60, 70, 80, 90, and 100. The graph shows a huge fluctuation in the loss with the increase in the number of rounds. The highest loss was identified at round 100 as 0.7711, whereas the lowest loss was identified at round 40 as 0.7051.

Fig. (**67**) represents the computational time taken by various rounds. The highest computational time has been taken by round 1 at 11.03 minutes. However, the lowest computational time was taken by round 26, 0.19 minutes. Furthermore, a huge fluctuation in the computational time with respect to the number of rounds has been identified.

Fig. (**68**) illustrates the computational time taken by various rounds at a difference of 10. This predicts that the highest computation time has been taken by round 90 at 7.08 minutes and the lowest by round 10 at 2.45 minutes.

Figs. (**69**-**73**) show the graph of computational time with respect to number of rounds varying from 1-20, 21-40, 41-60, 61-80, and 81-100, respectively for training the proposed FedPneu model with five clients architecture. The graphs show that the highest computational time was taken by round 1 at 11.03 minutes. However, the lowest time was taken by round 26 at 0.19 minutes.

Fig. (**74**) illustrates the computational time taken by rounds 1-20, 21-40, 41-60, 61-80, and 81-100 to predict pneumonia using the proposed FedPneu model. The highest computational time was taken by rounds 41-60, which was 113.16 minutes, whereas the least computation time was taken by rounds 21-40, which was 61.17 minutes. This identifies that training rounds 21-40 have resulted in a short duration in comparison to rounds 1-20, 41-60, 61-80, and 81-100.

The proposed FL framework with 2, 3, 4, and 5 client architectures has provided the highest accuracies of 85.632%, 85.536%, 76.112%, and 74.123%, respectively. It has been observed that the 2 client architecture has outperformed in comparison to the 3, 4, and 5-client federated frameworks for pneumonia detection. In the performance analysis of these frameworks, it has also been identified that the increase in the number of clients has enhanced the computational time. Further, the accuracy has also started to decrease with the increase in the client value. Though, the overfitting has not been observed in the proposed FL-based framework, however, it is a significant concern in the deep learning architectures if the data is not balanced. Overfitting of the model creates a scenario, where the model out-performs the training data but underperforms for the unseen data. The FL architecture reduces the risk of overfitting due to its behavior of working in a decentralized framework. However, if the model provides the overfitted results with the inconsistent and imbalanced dataset, the aggregation technique could be changed from FedAVG to FedProx, which works better with the heterogeneous spread of the dataset over numerous clients. In addition, to avoid the overfitted model, the dataset could be enhanced by performing data augmentation and implementing the model with various federated hyperparameter tuning techniques.

### 5.5 Performance Comparison of the Proposed FedPneu Model with the State-of-the-art Models

This subsection depicts the comparative analysis of the proposed model and the state-of-the-art models in terms of global accuracy.

Fig (**75**) depicts a comparative graph of the proposed FedPneu model with the other existing models. In ( 40 ), the authors predicted COVID-19 pneumonia using the FedNova aggregation technique, and the accuracy of the results was identified as 5.57%, which is very low. Similarly, in ( 35 ), authors have implemented an FL framework by using variants of transfer learning and CNN as the base models. However, the results have depicted that the FL implemented with CNN, ResNet50, and AlexNet has shown an accuracy of 74%, 73%, and 73%, respectively. Additionally, one more work presented in ( 34 ) has implemented a FedHealth model for Parkinson's disease prediction, which has shown an outcome of 74.90% accuracy, which is less as compared to the proposed FedPneu model. This comparison results show that the proposed FedPneu model outperforms by resulting in better global accuracy values when implemented with 100 rounds and two, three, four, and five client architecture.

Table **9** shows a performance description of state-of-the-art models. In addition, the base model and problem domain of the proposed work have also been stated.

The results of the proposed model have shown that the prediction of pneumonia in grey-scale CXRs is a difficult task. Pneumonia is detected by looking at the white patches in the CXRs. However, these images are grey-scale, which makes it difficult to differentiate the disease and the lungs. Hence, the proposed FedPneu model has been implemented with a DL-based model for the early prediction of pneumonia. This model extracts essential features in the CXRs by using hidden layers. Additionally, the proposed model with 2, 3, 4, and 5 clients architecture has shown an outperforming global accuracy of 85.632%, 85.536%, 76.112%, and 74.132%, respectively as presented in Table **9**.

## CONCLUSION

Pneumonia has become the major cause of rising mortality rates across the globe. The proposed work uses a FL framework to develop a privacy-protected, computationally efficient, and optimal pneumonia prediction model. The FL is a distributed architecture that uses DL to train multiple participating clients. It transmits the base model to the clients rather than sending the patient's private data to the central server. The proposed FedPneu federated deep learning model has been developed by configuring the hyperparameters, such as learning rate, batch size, optimizer, momentum, and decay, have been kept as 0.0001, 32, SGD, 0.9, and 0.000001 for the base classifier, respectively, and the key parameters for the proposed federated deep learning framework have been configured as 2, 3, 4, and 5 clients, 10 epochs, 100 rounds, and random split as 42. The results of the proposed federated deep learning-based FedPneu model have been demonstrated in terms of global accuracy, global loss, and computational time to train the model. The model implemented in two, three, four, and five client architecture has achieved an overall accuracy of 85.632%, 85.536%, 76.112%, and 74.132%, respectively. The computational time to perform the complete implementation of the proposed privacy-protected FedPneu model has been computed as the highest in 4-client architecture. Further, the performance comparison of the proposed FedPneu model in terms of global accuracy with the existing models has also been done, which showed that the proposed FedPneu model out-performs and results in the highest global accuracy. The proposed model is highly efficient and privacy protected and developed in a muti-silo framework with the IID data distribution. In the future, the transfer learning models can be implemented as the base classifiers in an FL-architecture for pneumonia prediction to achieve higher accuracy in each round and improve the time complexity of the model.

## Figures and Tables

**Fig. (1):**
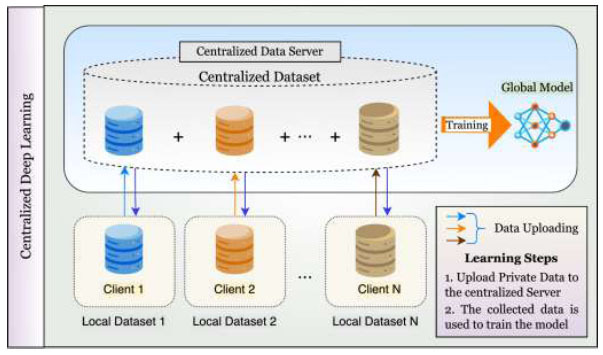
A systematic framework of centralized deep learning.

**Fig. (2):**
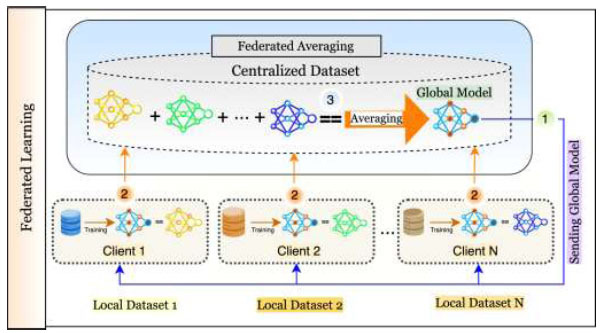
A systematic framework of distributed federated learning.

**Fig. (3):**
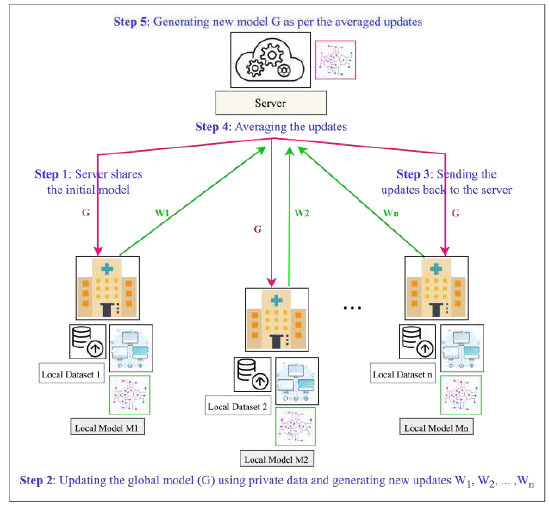
A schematic architecture of federated learning-based disease prediction model.

**Fig. (4):**
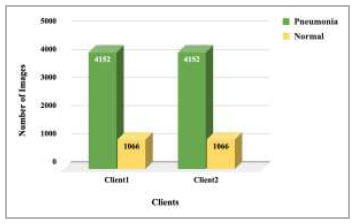
Data distribution for training each client in 2 clients' FL architecture.

**Fig. (5):**
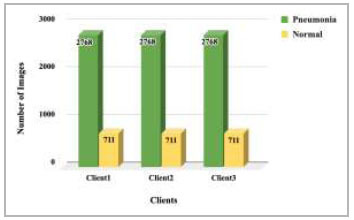
Data distribution for training each client in 3 clients’ FL architecture.

**Fig. (6):**
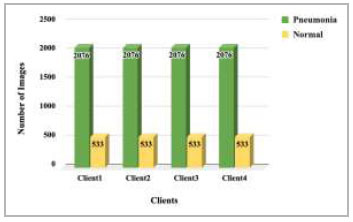
Data distribution for training each client in 4 clients’ FL architecture.

**Fig. (7):**
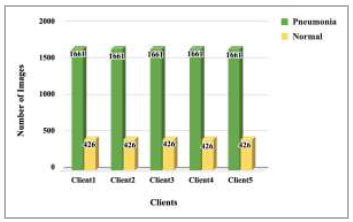
Data distribution for training each client in 5 clients’ FL architecture .

**Fig. (8):**
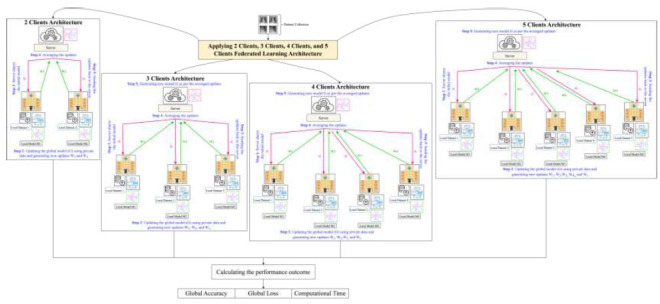
The schematic diagram of the proposed FL approach with 2 clients, 3 clients, 4 clients, and 5 clients’ architecture.

**Fig. (9):**
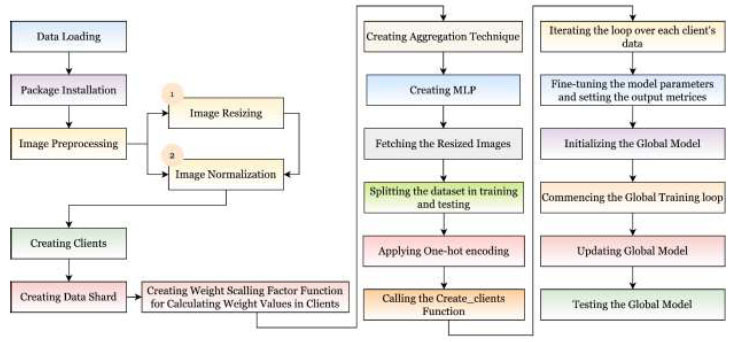
Building steps of the proposed FL framework for pneumonia detection.

**Fig. (10):**
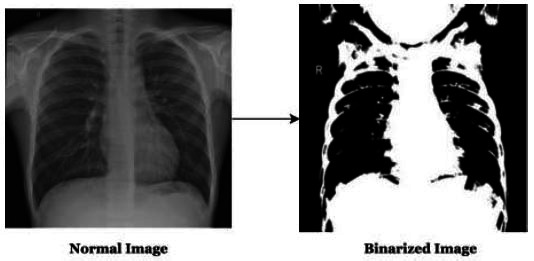
Binarizing of the pneumonia CXR.

**Fig. (11):**
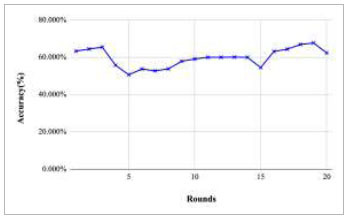
Performance analysis of FedPneu model with two clients in terms of global accuracy in rounds 1-20.

**Fig. (12):**
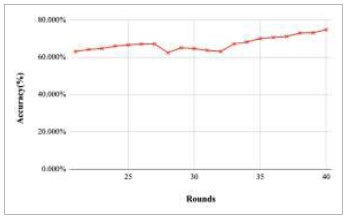
Performance analysis of FedPneu model with two clients in terms of global accuracy in rounds 21-40.

**Fig. (13):**
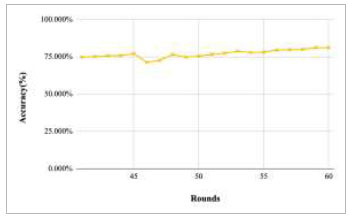
Performance analysis of FedPneu model with two clients in terms of global accuracy in rounds 41-60.

**Fig. (14):**
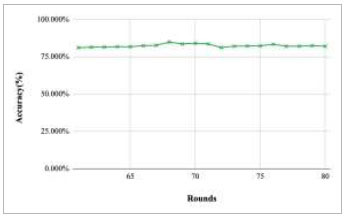
Performance analysis of FedPneu model with two clients in terms of global accuracy in rounds 61-80.

**Fig. (15):**
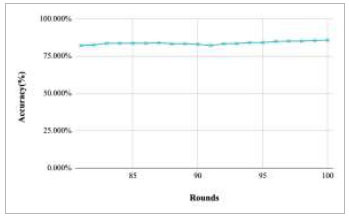
Performance analysis of FedPneu model with two clients in terms of global accuracy in rounds 81-100.

**Fig. (16):**
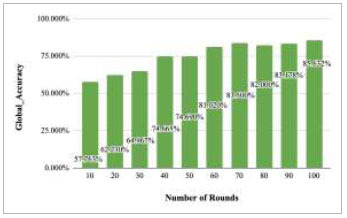
Performance measure of proposed FedPneu model with two clients in terms of global accuracy after each 10 rounds.

**Fig. (17):**
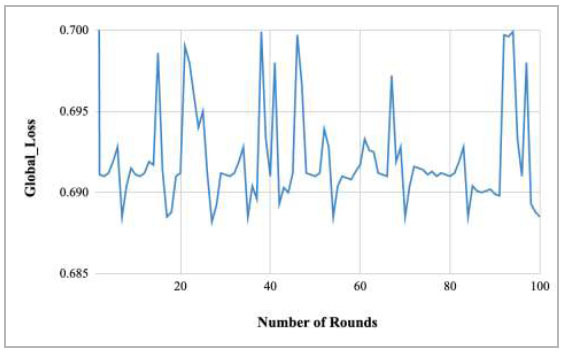
Performance analysis of the proposed FedPneu model with two clients in terms of global loss with respect to the number of rounds.

**Fig. (18):**
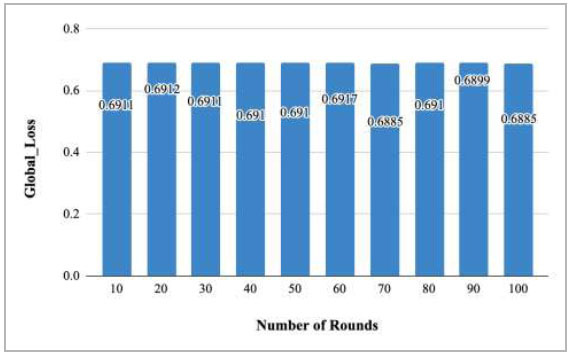
Performance measure of proposed FedPneu model with two clients in terms of global loss after each 10 rounds.

**Fig. (19):**
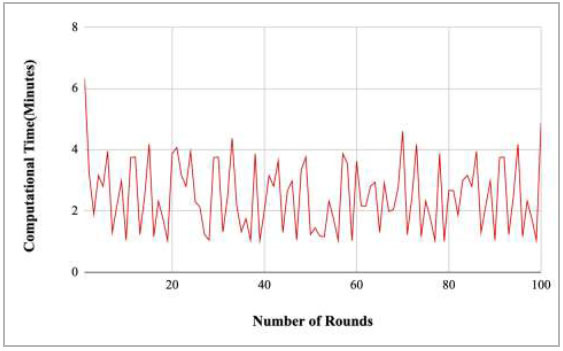
Performance analysis of the proposed FedPneu model with two clients in terms of computational time with respect to a number of rounds.

**Fig. (20):**
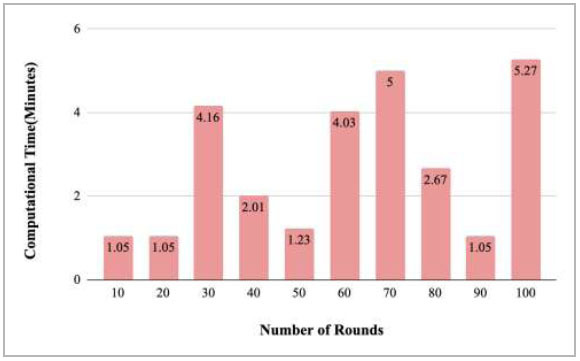
Performance measure of proposed FedPneu model with two clients in terms of computation time after each 10 rounds.

**Fig. (21):**
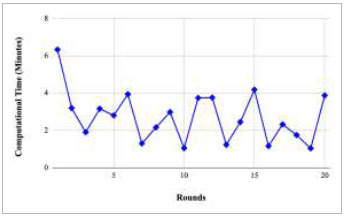
Performance analysis of the FedPneu model with two clients in terms of computational time in rounds 1-20.

**Fig. (22):**
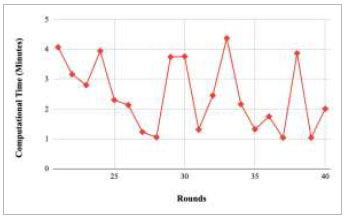
Performance analysis of the FedPneu model with two clients in terms of computational time in rounds 21-40.

**Fig. (23):**
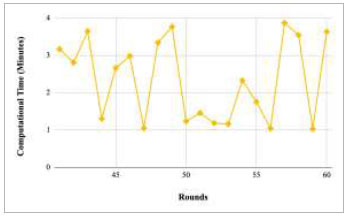
Performance analysis of the FedPneu model with two clients in terms of computational time in rounds 41-60.

**Fig. (24):**
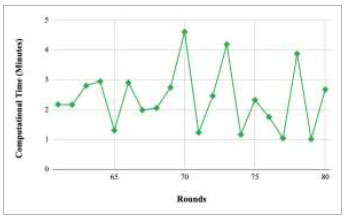
Performance analysis of the FedPneu model with two clients in terms of computational time in rounds 61-80.

**Fig. (25):**
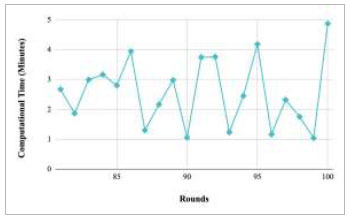
Performance analysis of the FedPneu model with two clients in terms of computational time in rounds 81-100.

**Fig. (26):**
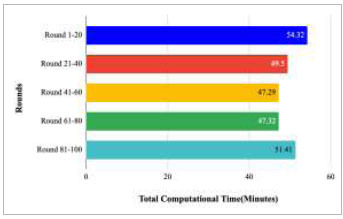
Total computational time taken by rounds 1-20, 21-40, 41-60, 61-80, and 81-100 to implement the proposed FedPneu model with two clients.

**Fig. (27):**
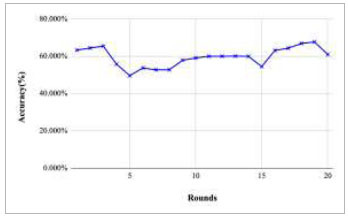
Performance analysis of the FedPneu model with three clients in terms of global accuracy in rounds 1-20.

**Fig. (28):**
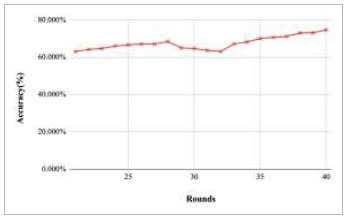
Performance analysis of the FedPneu model with three clients in terms of global accuracy in rounds 21-40.

**Fig. (29):**
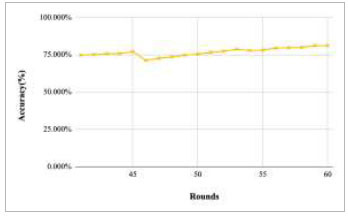
Performance analysis of the FedPneu model with three clients in terms of global accuracy in rounds 41-60.

**Fig. (30):**
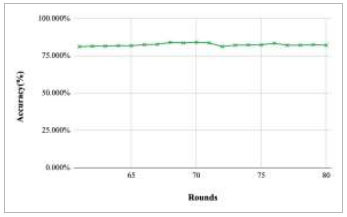
Performance analysis of the FedPneu model with three clients in terms of global accuracy in rounds 61-80.

**Fig. (31):**
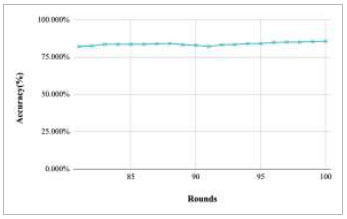
Performance analysis of the FedPneu model with three clients in terms of global accuracy in rounds 81-100.

**Fig. (32):**
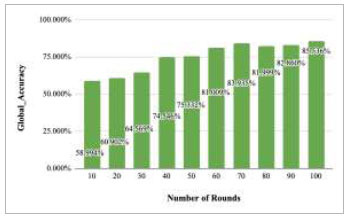
Performance measure of the proposed FedPneu model with three clients in terms of global accuracy after each 10 rounds.

**Fig. (33):**
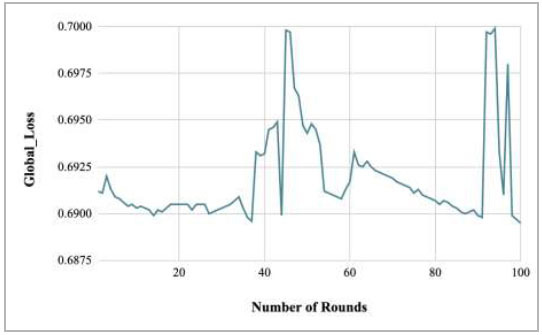
Performance analysis of the FedPneu model with three clients in terms of global loss with respect to the number of rounds.

**Fig. (34):**
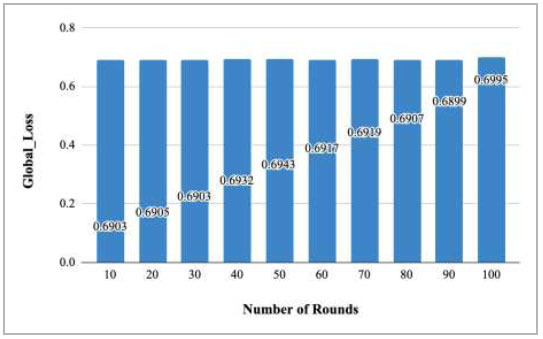
Performance measure of proposed FedPneu model with three clients in terms of global loss after each 10 rounds.

**Fig. (35):**
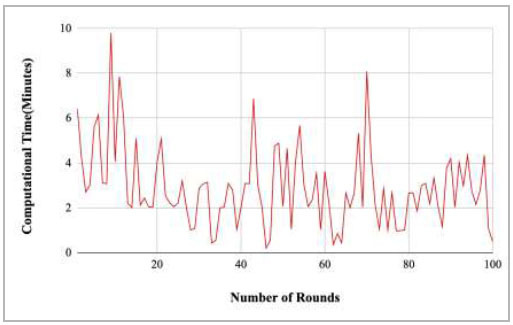
Performance analysis of the proposed FedPneu model with three clients in terms of computational time with respect to the number of rounds.

**Fig. (36):**
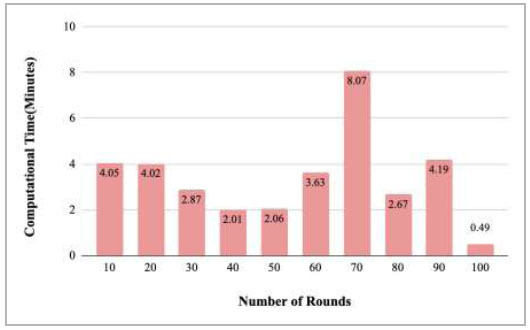
Performance measure of proposed FedPneu model with three clients in terms of computation time after each 10 rounds.

**Fig. (37):**
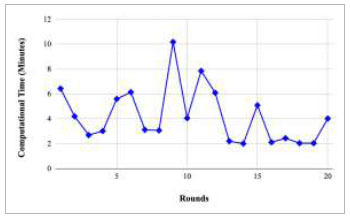
Performance analysis of the proposed FedPneu model with three clients in terms of computational time in rounds 1-20.

**Fig. (38):**
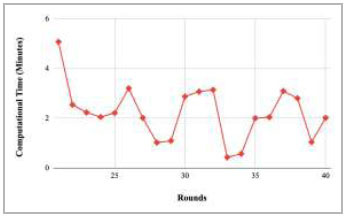
Performance analysis of the proposed FedPneu model with three clients in terms of computational time in rounds 21-40.

**Fig. (39):**
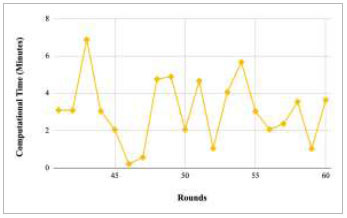
Performance analysis of the proposed FedPneu model with three clients in terms of computational time in rounds 41-60.

**Fig. (40):**
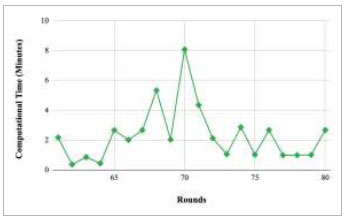
Performance analysis of the FedPneu model with three clients in terms of computational time in rounds 61-80.

**Fig. (41):**
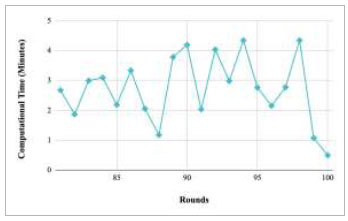
Performance analysis of the proposed FedPneu model with three clients in terms of computational time in rounds 81-100.

**Fig. (42):**
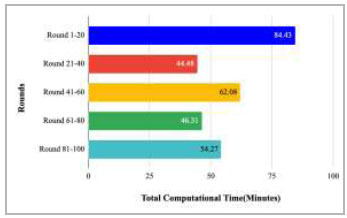
Total computational time taken by rounds 1-20, 21-40, 41-60, 61-80, and 81-100 to implement the proposed FedPneu model with three clients.

**Fig. (43):**
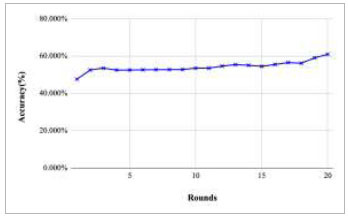
Performance analysis of the proposed FedPneu model with four clients in terms of global accuracy in rounds 1-20.

**Fig. (44):**
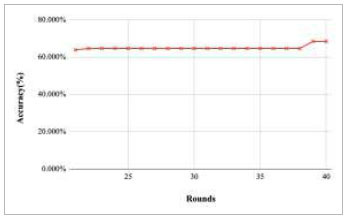
Performance analysis of the FedPneu model with four clients in terms of global accuracy in rounds 21-40.

**Fig. (45):**
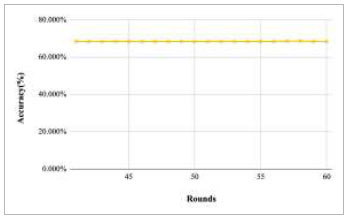
Performance analysis of the proposed FedPneu model with four clients in terms of global accuracy in rounds 41-60.

**Fig. (46):**
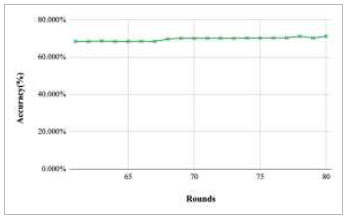
Performance analysis of the proposed FedPneu model with four clients in terms of global accuracy in rounds 61-80.

**Fig. (47):**
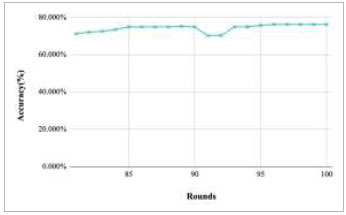
Performance analysis of the proposed FedPneu model with four clients in terms of global accuracy in rounds 81-100.

**Fig. (48):**
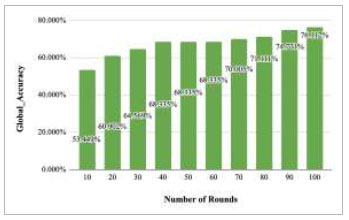
Performance measure of the proposed FedPneu model with four clients in terms of global accuracy after each 10 rounds.

**Fig. (49):**
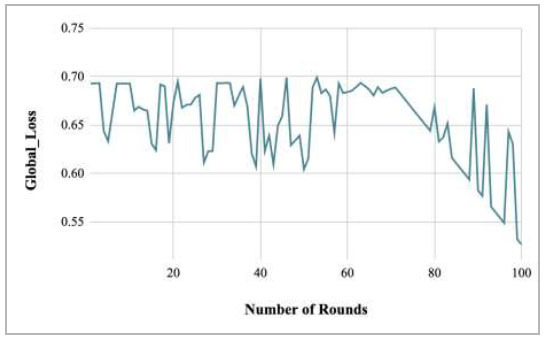
Performance analysis of the proposed FedPneu model with four clients in terms of global loss with respect to the number of rounds.

**Fig. (50):**
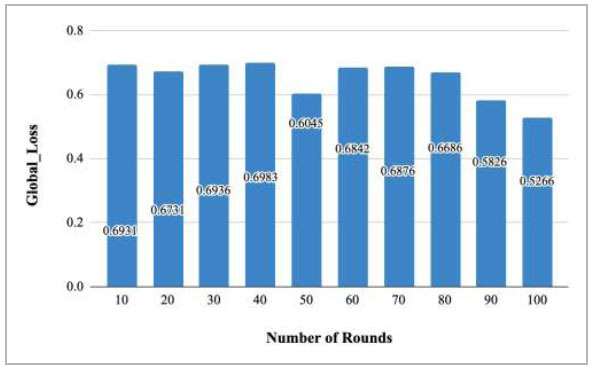
Performance measure of the proposed FedPneu model with four clients in terms of global loss after each 10 rounds.

**Fig. (51):**
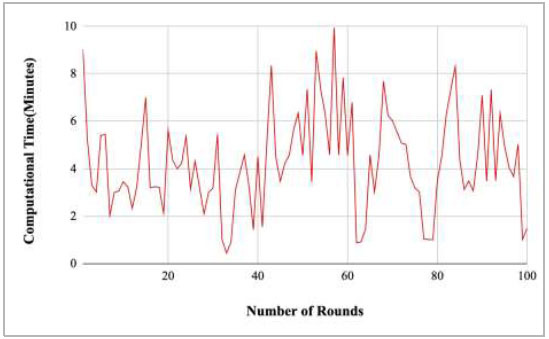
Performance analysis of the proposed FedPneu model with four clients in terms of computational time with respect to the number of rounds.

**Fig. (52):**
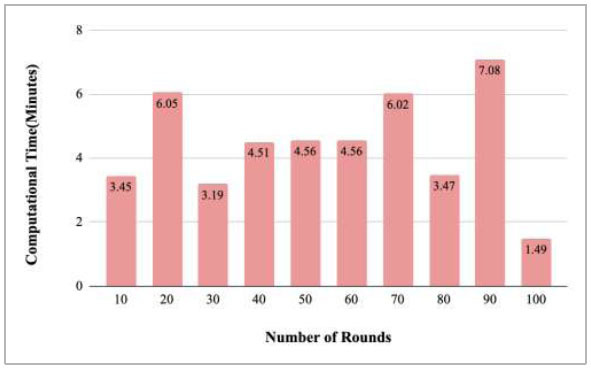
Performance measure of the proposed FedPneu model with four clients in terms of computation time after each 10 rounds.

**Fig. (53):**
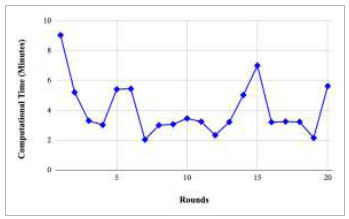
Performance analysis of the proposed FedPneu model with four clients in terms of computational time in rounds 1-20.

**Fig. (54):**
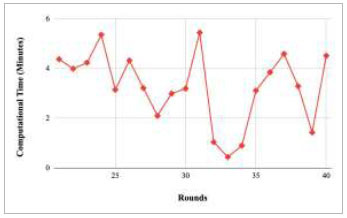
Performance analysis of the proposed FedPneu model with four clients in terms of computational time in rounds 21-40.

**Fig. (55):**
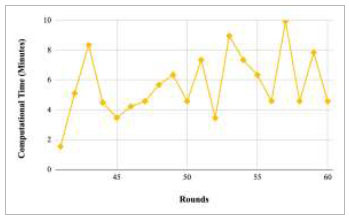
Performance analysis of the proposed FedPneu model with four clients in terms of computational time in rounds 41-60.

**Fig. (56):**
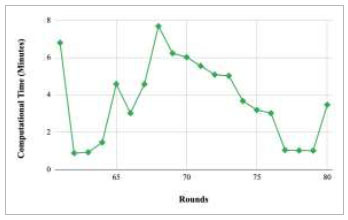
Performance analysis of the proposed FedPneu model with four clients in terms of computational time in rounds 61-80.

**Fig. (57):**
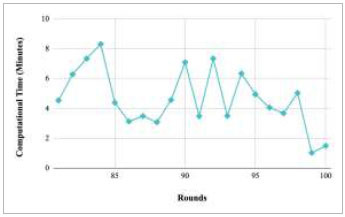
Performance analysis of the proposed FedPneu model with four clients in terms of computational time in rounds 81-100.

**Fig. (58):**
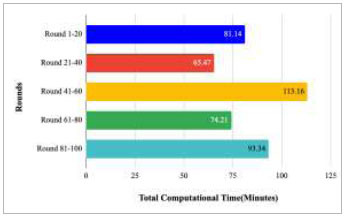
Total computational time taken by rounds 1-20, 21-40, 41-60, 61-80, and 81-100 to implement the proposed FedPneu model with four clients.

**Fig. (59):**
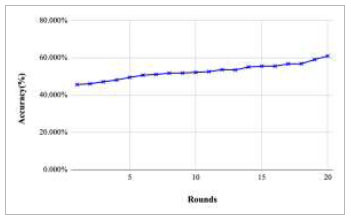
Performance analysis of the proposed FedPneu model with five clients in terms of global accuracy in rounds 1-20.

**Fig. (60):**
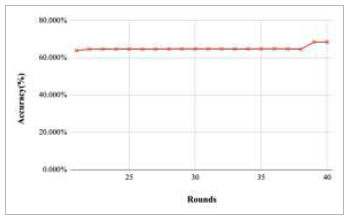
Performance analysis of the proposed FedPneu model with five clients in terms of global accuracy in rounds 21-40.

**Fig. (61):**
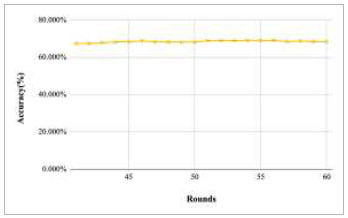
Performance analysis of the proposed FedPneu model with five clients in terms of global accuracy in rounds 41-60.

**Fig. (62):**
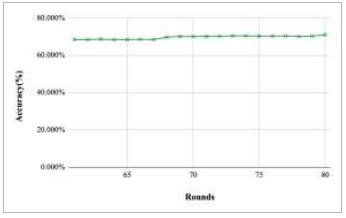
Performance analysis of the proposed FedPneu model with five clients in terms of global accuracy in rounds 61-80.

**Fig. (63):**
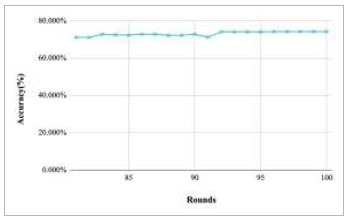
Performance analysis of the proposed FedPneu model with five clients in terms of global accuracy in rounds 81-100.

**Fig. (64):**
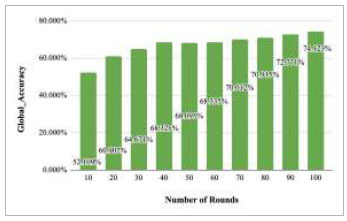
Performance measure of the proposed FedPneu model with five clients in terms of global accuracy after each 10 rounds.

**Fig. (65):**
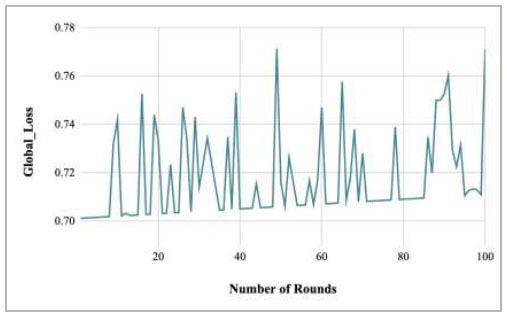
Performance analysis of the proposed FedPneu model with five clients in terms of global loss with respect to the number of rounds.

**Fig. (66):**
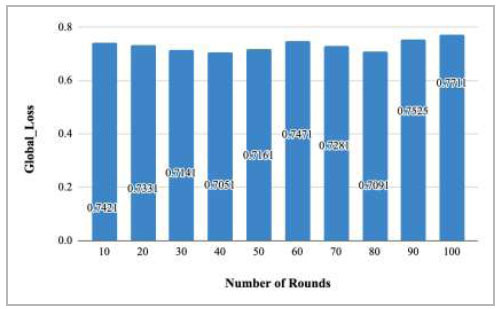
Performance measure of the proposed FedPneu model with five clients in terms of global loss after each 10 rounds.

**Fig. (67):**
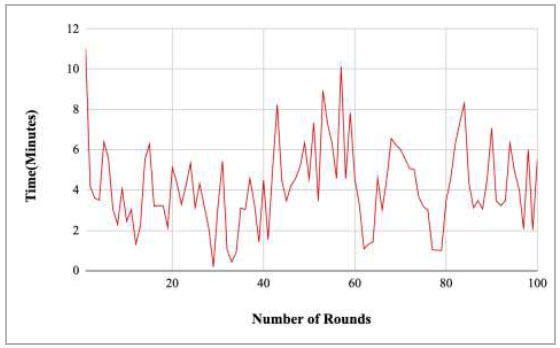
Performance analysis of the proposed FedPneu model with five clients in terms of computational time with respect to the number of rounds.

**Fig. (68):**
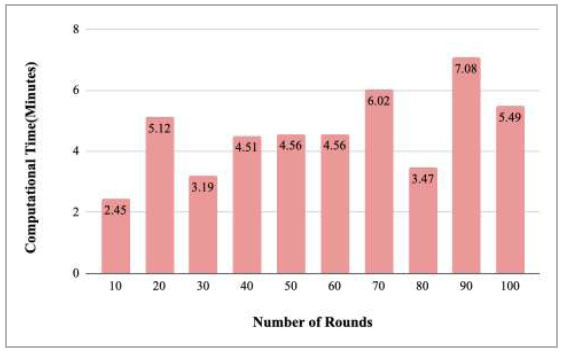
Performance measure of the proposed FedPneu model with five clients in terms of computation time after each 10 rounds.

**Fig. (69):**
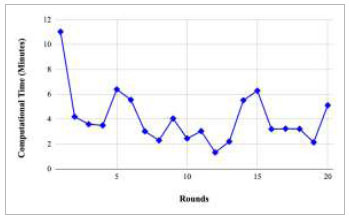
Performance analysis of the proposed FedPneu model with five clients in terms of computational time in rounds 1-20.

**Fig. (70):**
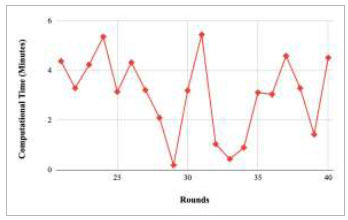
Performance analysis of the proposed FedPneu model with five clients in terms of computational time in rounds 21-40.

**Fig. (71):**
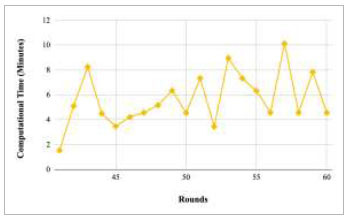
Performance analysis of the proposed FedPneu model with five clients in terms of computational time in rounds 41-60.

**Fig. (72):**
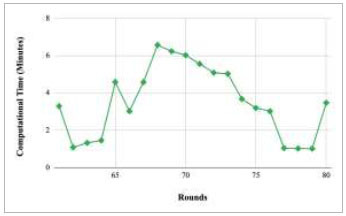
Performance analysis of the proposed FedPneu model with five clients in terms of computational time in rounds 61-80.

**Fig. (73):**
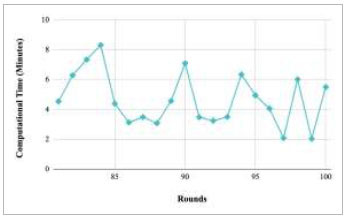
Performance analysis of the proposed FedPneu model with five clients in terms of computational time in rounds 81-100.

**Fig. (74):**
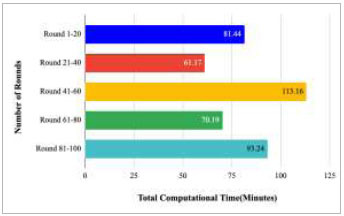
Total computational time taken by rounds 1-20, 21-40, 41-60, 61-80, and 81-100 to implement the proposed FedPneu model with five clients.

**Fig. (75):**
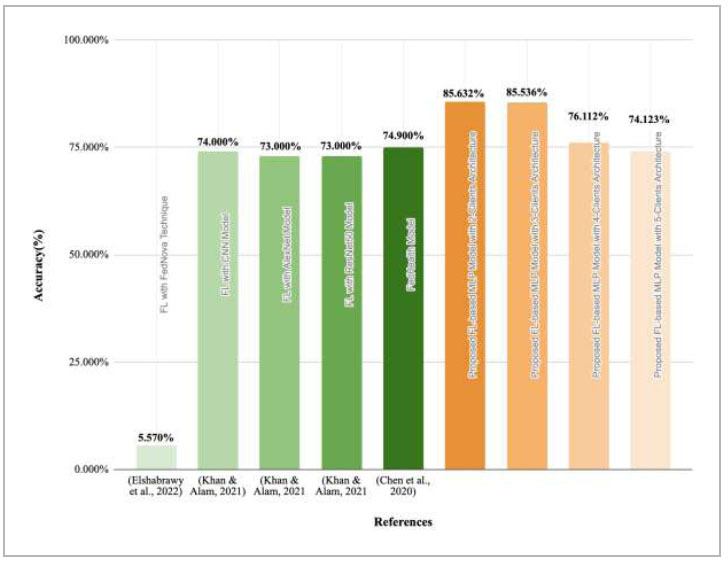
Performance comparison of the proposed FedPneu model with the state-of-the-art models.

**Table 1:** Difference between deep learning and federated learning.

**Characteristics**	**Deep Learning**	**Federated Learning**
**Architecture**	Centralized	Distributed
**Focus**	Focuses on the centralized training of the model to achieve improved performance.	Focuses on the collaborative training of models across multiple decentralized clients while maintaining data privacy.
**Data Handling**	Centralized data is kept in a single location.	IID and non-IID distributed data
**Privacy**	Doesn’t inherently address privacy concerns.	Inherently addresses data privacy concerns.
**Bandwidth**	Requires high bandwidth to send the data to the server.	Requires lowered bandwidth as only the trained models are sent to the server.
**Flexibility**	Limited flexibility	Highly flexible
**Application**	It is powerful for tasks involving recognizing patterns from unstructured data. ***i.e*.** image classification, autonomous vehicle, and natural language processing.	It is particularly useful in scenarios, where, data confidentiality, privacy, and access rights are a concern. ***i.e*.,** healthcare and finance.

**Table 2:** A detailed summary of the various existing deep learning models trained using federated learning.

**Refs.**	**Base Model**	**Hyperparameters**	**Performance** **Outcome**	**Area**	**Research gaps**
[25]	FaNet using CNN	Epoch: 1000, Optimizer: Adam, Learning rate: 0.01	98.28%	Pneumonia	The dataset used for the implementation is centralized, which is highly privacy-sensitive.
[26]	ChexNet model using CNN architecture	Batch size: 16, Optimizer: Adam, Learning rate: 0.01	0.435: F1-score	Pneumonia	The model achieved a smaller value of the F1-score, which reflects that the model is not optimal for detecting pneumonia efficiently.
[27]	VGG19, InceptionResNetV2, InceptionV3, DenseNet201, VGG16, Xception, MobileNetv2 ResNet50	Epoch: 300, Batch size: 32, Optimizer: Adam, Learning rate: 0.00001	ResNet50: 96.61%	Pneumonia	The models implemented by the authors have significantly improved the accuracy. However, the configured learning rate is small, which may lead to higher computational time for the model to achieve optimal accuracy.
[28]	AlexNet model	Epoch: 20, Optimizer: SGD, Learning rate: 0.0001	94.43%	Pneumonia and COVID-19	The model is trained with a small dataset, which may result in overfitting challenges.
[29]	InceptionV3	Epoch: 20 Optimizer: SGD	97%	Pneumonia	The authors provided limited information on the hyperparameter configuration.
[30]	ResNet18, COVID-Net, ResNext, MobileNet	Clients: 5, Epoch: 3, Learning rate: 0.00002, Round: 100, Weight decay: 0.0000001, Batch size: 10	Not Mentioned	COVID-19 pneumonia	The authors have employed an exceedingly large value of learning rate, which could potentially lead to a delay in achieving desired performance outcomes.
[31]	COVID- CAPS	Not mentioned	Accuracy: 95.7% Sensitivity: 90% Specificity: 95.8% AUC: 0.97	COVID-19	The COVID-CAPS model introduced by the authors has been applied to the NIH chest X-ray dataset. Nevertheless, the presence of 13 additional diseases in the dataset, apart from COVID-19, constrains in achieving the potential outcomes of the proposed research to diagnose COVID-19.
[32]	InceptionV3	Loss function: Cross entropy, Epoch: 20, Optimizer: Adam, Learning rate: 0.001, Decay rate: 0.96	AUC:1	COVID-19 and pneumonia	The authors have provided the outcome in AUC as 1, which is not justifiable in the practical scenarios.
[33]	MobileNetV2, ResNet18, DenseNet121, and ResNet50	Clients: 3, Epochs: 10, Batch size: 16, Rounds: 5, Optimizer: Adam, SGD+momentum, and Adamx, Learning rate: 0.001,	Accuracy: 87.3%	Pneumonia	The authors have omitted the details regarding the hyperparameter settings for the learning rate in their proposed work.
[34]	CNN	Clients:5, Learning rate: 0.01, Batch size: 64, Epochs: 80	Accuracy: 99.4%	Parkinson’s	The authors have trained the CNN model for a large number of epochs which results in increasing the computation time and overhead.
[35]	CNN, ResNet50, AlexNet, VGG16	Clients: 2 Batch size: 1, Epochs: 50, Learning rate: 0.000001	Accuracy: CNN: 74% ResNet50: 73% AlexNet: 73% VGG16: 91%	Pneumonia	The authors have used a very small value of batch size. The model can be enhanced and the performance can be measured by increasing the value of batch size.
[36]	ResNext50, convNet, AlexNet, ResNet18	Learning rate: 0.0001 Optimizer: Adam and SGD, Aggregation: FedAVG, COMED, and GEOMED	Accuracy with GEOMED: ResNext50: 77% convNet: 81%, AlexNet: 86%, ResNet18: 82%	Pneumonia	The value of the learning rate has been taken very small, which may result in increasing resource utilization.
[37]	ResNet50, VGG16	Clients: 4, Learning rate: 0.001, Epoch: 10, Batch size: 2	Accuracy: ResNet50: 97%, VGG16: 94.40%	COVID-19 pneumonia	The authors have omitted the information on various hyperparameters, namely optimizer, aggregation methods, and rounds, which creates difficulty in understanding the building blocks of the model architecture.
[38]	RetinaNet	Clients: 3, Optimizer: Adam, Learning rate: 0.0001	Not Mentioned	COVID-19	The authors did not include details on the hyperparameters, namely, rounds, aggregation methods, and batch size.
[39]	VGG16	Round: 10, Aggregation: FedAVG, Epoch: 15, Optimizer: Adam, and Learning rate: 0.001	Accuracy: VGG16: 79.32%	COVID-19	The authors employed a dataset comprising only 2230 images, which is too limited to optimize model training adequately.
[40]	FL architecture	Client: 4, Round: 10, Learning rate: 0.01, Batch size: 64, Aggregation: FedAVG, FedProx, FedNOVA, SCAFFOLD, FedBN	Accuracy: FedAVG: 79.58%, FedProx: 76.92%, FedNOVA: 5.57%, SCAFFOLD: 79.18%, FedBN: 84.4%	COVID-19 pneumonia	The authors have chosen to train the model using a small dataset. However, this restricted dataset size gives rise to challenges related to overfitting.
[41]	Particle Swarm Optimization	Client: 10, Round: 10, Optimizer: Adam, Learning rate: 0.02, Epoch: 30, Aggregation: FedAVG	Accuracy for COVID-19: 96.15%, Accuracy for pneumonia: 96.55%	COVID-19 pneumonia	The authors have employed a substantial number of clients with a dataset comprising only 5856 images. The division of the dataset into 10 clients for model training inadvertently results in insufficient training, thereby giving rise to overfitting issues.
[42]	DMFL_Net with DenseNet169	Not Mentioned	Accuracy: 98.45%	COVID-19 pneumonia, cancer, tuberculosis	The authors have not provided a detailed description of hyperparameters which poses a challenge to the other researchers seeking insights into the model's architectural construction.

**Table 3:** A description of various packages and library installations for optimal federated learning framework creation for pneumonia classification.

**Packages**	**Package**	**Package**	**Package**
numpy	pandas	random	cv2
os	tqdm	train_test_split	LabelBinarizer
shuffle	accuracy_score	tensorflow	expand_dims
Sequential	Conv2D	Input	Lambda
MaxPooling2D	Activation	Flatten	Dense
SGD	backend	paths	scikit-learn

**Table 4:** A detailed description of evaluation parameters and hyperparameters used during the development of the federated learning model for pneumonia detection.

**Parameters**	**Naming Mentions**	**Value**	**Description**
Clients	*E_ser_*	3, 4	Total number of hospitals participating in the FL architecture
Batch size	B	32	Before the model is updated, how many numbers of samples are processed
Learning rate	λ	0.0001	The learning rate has been used for identifying the value of step size in each iteration
Momentum	β	0.9	To build the inertia towards the direction of the search space
Rounds	R	100	Total number of rounds used for computing model outcomes
Optimizer	SGD	1	It is used for adjusting the FedPneu model parameters such as biases and weights to result in an optimal performance
Performance metric	Accuracy, loss, and computational time	-	To predict the performance outcome of the FedPneu model
Epochs	E	10	It shows the number of iterations of all training data at the client side in one cycle
Output classes	Pneumonia and Normal	2	NA
Train: Test ratio	NA	90:10	NA
Input image size	NA	224*224	To be substituted to the FedPneu model
Number of images	NA	10440	The number of images was divided among different numbers of clients
random_split	NA	42	It has been used for controlling the shuffling process in the train_test_split() function

**Table 5:** Computed global loss of the proposed FedPneu model with two clients after each of 10 rounds starting from round 5.

**Rounds**	**Global Loss**	**Rounds**	**Global Loss**
5	0.6919	55	0.6904
15	0.6986	65	0.6911
25	0.695	75	0.6911
35	0.6885	85	0.6904
45	0.6912	95	0.6933

**Table 6:** Computed global loss of the proposed FedPneu model with three clients after each of 10 rounds starting from round 5.

**Rounds**	**Global Loss**	**Rounds**	**Global Loss**
5	0.6909	55	0.6911
15	0.6902	65	0.6925
25	0.6905	75	0.6911
35	0.6903	85	0.6903
45	0.6998	95	0.6933

**Table 7:** Computed global loss of the proposed FedPneu model with four clients after each 10 rounds starting from round 5.

**Rounds**	**Global Loss**	**Rounds**	**Global Loss**
5	0.6334	55	0.687
15	0.631	65	0.6867
25	0.6783	75	0.6666
35	0.6803	85	0.6106
45	0.6593	95	0.5546

**Table 8:** Computed global loss of the proposed FedPneu model with five clients after each of 10 rounds starting from round 5.

**Rounds**	**Global Loss**	**Rounds**	**Global Loss**
5	0.7016	55	0.7066
15	0.7026	65	0.7576
25	0.7036	75	0.7086
35	0.7046	85	0.7096
45	0.7056	95	0.7106

**Table 9:** A comparative analysis of the proposed model with the state-of-the-art models along with their problem domain.

**Refs.**	**Model/** **Framework**	**Dataset**	**Accuracy**	**Problem Domain**
(40)	Federated Learning	Dataset1: 188 images, Dataset 2: 98 images, Dataset 3: 317 images, Dataset 4: 5840 images	FedNova= 5.57%	COVID-19 pneumonia detection
(35)	Federated Learning	5856 images	CNN= 74%	Pneumonia prediction
(35)	Federated Learning	5856 images	AlexNet= 73%	Pneumonia prediction
(35)	Federated Learning	5856 images	ResNet50= 73%	Pneumonia prediction
(34)	FedHealth	The dataset has been collected in real-time from 130 patients aged between 19 to 87 years	CNN= 74.9%	Parkinson’s disease detection
Proposed Model with 2 Clients Architecture		10,440 CXR images	FedPneu = 85.632%	Pneumonia prediction
Proposed Model with 3 Clients Architecture			FedPneu = 85.536%	Pneumonia prediction
Proposed Model with 4 Clients Architecture			FedPneu = 76.112%	Pneumonia prediction
Proposed Model with 5 Clients Architecture			FedPneu = 74.132%	Pneumonia prediction

## Data Availability

The dataset used in this research is available at the following repositories: https://www.kaggle.com/datasets/paultimothymooney/chest-xray-pneumonia https://www.kaggle.com/code/pushkalpandey3/pneumonia-binary-classification/data https://www.kaggle.com/datasets/parthachakraborty/pneumonia-chest-x-ray

## LIST OF ABBREVIATIONS

AI: Artificial Intelligence
ML: Machine Learning
DL: Deep learning
DNN: Deep Neural Networks
MRI: Medical Resonance Imaging
HIPAA: Health Insurance Portability and Accountability Act
MLP: Multi-layer Perceptron
